# Melanin-Like Nanomedicine in Photothermal Therapy Applications

**DOI:** 10.3390/ijms22010399

**Published:** 2021-01-01

**Authors:** Yale Yue, Xiao Zhao

**Affiliations:** 1 School of Basic Medical Sciences, Zhengzhou University, Zhengzhou 450001, China; yueyl2019@nanoctr.cn; 2 CAS Key Laboratory for Biomedical Effects of Nanomaterials and Nanosafety and CAS Center for Excellence in Nanoscience, National Center for Nanoscience and Technology of China, Beijing 100190, China; 3 Center of Materials Science and Optoelectronics Engineering, University of Chinese Academy of Sciences, Beijing 100049, China

**Keywords:** photothermal therapy, melanin-like nanoparticles, biocompatibility, photothermal conversion ability, tumor, bioimaging, clinical application

## Abstract

Photothermal therapy (PTT) mediated by nanomaterial has become an attractive tumor treatment method due to its obvious advantages. Among various nanomaterials, melanin-like nanoparticles with nature biocompatibility and photothermal conversion properties have attracted more and more attention. Melanin is a natural biological macromolecule widely distributed in the body and displays many fascinating physicochemical properties such as excellent biocompatibility and prominent photothermal conversion ability. Due to the similar properties, Melanin-like nanoparticles have been extensively studied and become promising candidates for clinical application. In this review, we give a comprehensive introduction to the recent advancements of melanin-like nanoparticles in the field of photothermal therapy in the past decade. In this review, the synthesis pathway, internal mechanism and basic physical and chemical properties of melanin-like nanomaterials are systematically classified and evaluated. It also summarizes the application of melanin-like nanoparticles in bioimaging and tumor photothermal therapy (PTT)in detail and discussed the challenges they faced in clinical translation rationally. Overall, melanin-like nanoparticles still have significant room for development in the field of biomedicine and are expected to applied in clinical PTT in the future.

## 1. Introduction

**Figure 1:**
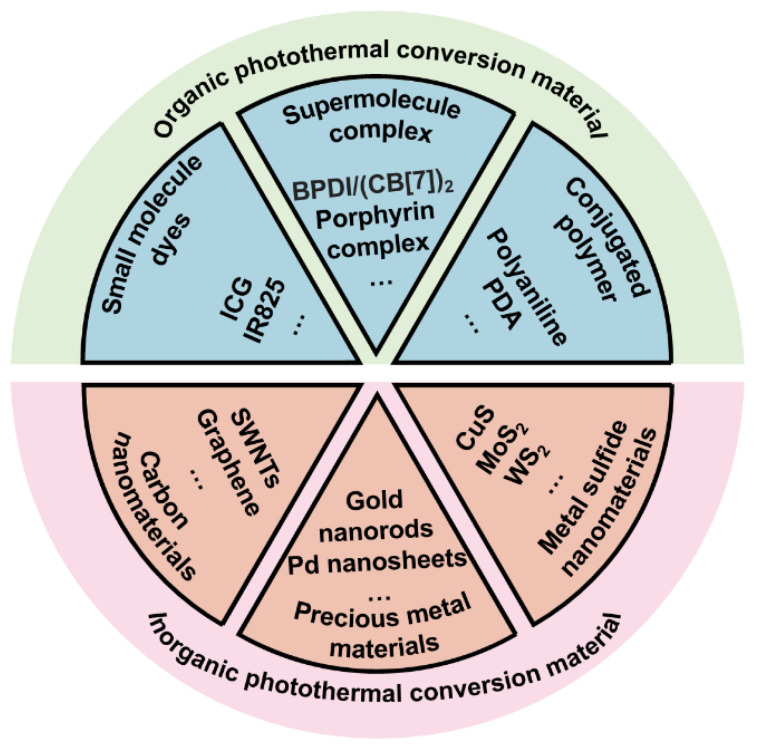
The classification of main photothermal conversion materials.

Cancer has been a major threat to human public health. According to estimates by the WHO (World Health Organization) in 2015, cancer has become the main cause of death before the age of 70 in 91 countries around the world [1]. Traditional cancer treatment methods such as surgery, radiotherapy and chemotherapy have reduced the burden of cancer to a certain extent, but severe systemic toxicity and multidrug resistance problems force people to seek new treatment methods [2,3]. Nanomaterials-mediated photothermal therapy (PTT) converts the absorbed light energy into heat through the photothermal conversion characteristic of nanocarrier, causing a rapid increase in local temperature. The constant high temperature (42–45 °C) for a certain period of time (15–60 min) will eventually ablates cancer cells. This thermal ablation therapy has become an alternative therapy for some unresectable solid tumors. In addition to the inherent advantages of photothermal therapy, nanomaterials-mediated PTT exhibits many other advantages, for example, it can be controlled in vivo and in vitro [4,5,6]. In vivo, the surface modification of nanoparticles can achieve targeted enrichment of photothermal conversion agents (PTCAs). In vitro, the laser power and near-infrared light (NIR) irradiation position can be controlled artificially. The controllability can reduce system toxicity and protect normal tissues from damage. As early as 2002, laser thermal ablation was reported for recurrent squamous cell carcinoma [7]. Recently, the tumor photoimmunotherapy proposed by Hisataka Kobayashi combines photodynamic therapy with immunotherapy and has entered clinical trials, greatly promoting the clinical translational research of PTT based on PTCAs [8,9,10]. A variety of materials can convert the absorbed near-infrared light into heat (Figure 1). According to the molecular structure, they can be systematically divided into organic and inorganic photothermal conversion materials [11,12,13].

Organic photothermal conversion materials exhibit good biocompatibility and can be divided into small molecule dyes, supramolecular complexes and conjugated polymers according to the molecular weight and polymerization method. Among them, indocyanine green (ICG) has been approved by the US Food and Drug Administration (FDA) for clinical imagine and has been widely used, confirming the safety of ICG in vivo [14,15,16]. However, severe photobleaching and instability limit the further applications of these photothermal conversion agents. Compared with small molecules and supramolecules, conjugated polymers such as polydopamine (PDA) have better stability and photothermal conversion efficiency (PCE). Moreover, abundant surface groups such as catechol make PDA easy to functionalize through Michael addition and Schiff reactions [17,18,19,20]. In addition to organic photothermal conversion materials, there are many inorganic nanomaterials that also exhibit excellent photothermal conversion properties. Some precious metal nanomaterials such as gold nanorods have local surface plasmon resonance (LSPR) effect which causes their temperature rise rapidly under laser irradiation. In addition, mature synthesis technology and surface functional modification make them promising photothermal materials [6,10,11]. Carbon nanomaterials and metal sulfide nanoparticles have also received a lot of attention in the research of tumor PTT due to their significant photothermal effects. Although inorganic nanomaterials exhibit amazing photothermal conversion capabilities, the high cost and poor biodegradability force researchers to develop safer and more efficient photothermal conversion materials [6]. Compared with synthetic inorganic materials, developing safe and efficient photothermal nanomaterials from natural components of organisms is more attractive.

**Figure 2:**
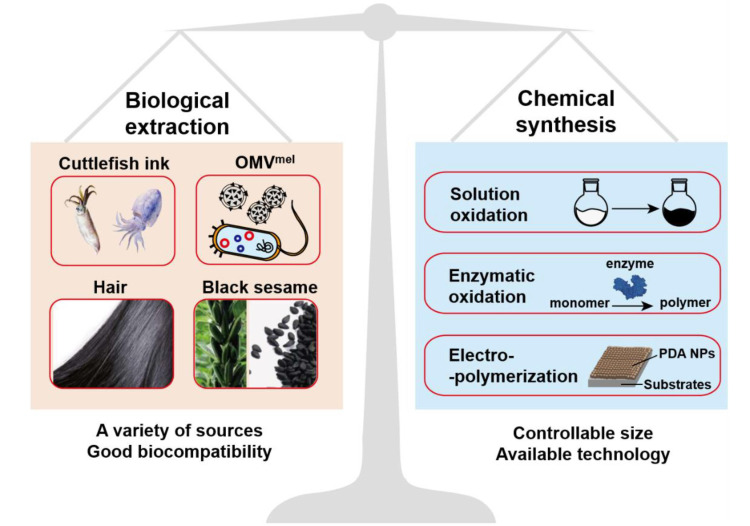
Schematic illustration of the synthesis method of melanin-like NPs.

Melanin is a natural polyphenol substance widely distributed in various organism such as hair and skin. It has been confirmed that melanin is formed by dopa and its derivatives through a series of oxidation reactions and coupling polymerization. The main components of melanin include 5,6-dihydroxyindole (DHI) and 5,6-dihydroxyindole-2-carboxylic acid (DHICA). The composition ratio of melanin is not static and it can be divided into dark eumelanin and red pheomelanin according to the absence (eumelanin) of sulfur in the composition or not (pheomelanin) [18,21]. As far as we know, there are no enzymes in the organism that can degrade natural melanin at the molecular level [22,23], but both nature sepia melanin and melanin-like nanoparticles exhibit a hydrogen peroxide-responsive decomposition behavior [20]. Because hydrogen peroxide is widespread in phagocytes, melanin and melanin-like nanoparticles show negligible toxicity in the body [24]. In addition, melanin exhibits many excellent physical and chemical properties including an obvious UV-vis absorption, outstanding photothermal conversion efficiency and other properties that contribute to its function [20]. The prominent features of melanin attract researchers to develop artificial melanin nanoparticles, also called melanin-like nanoparticles (melanin-like NPs). The ways for obtaining melanin-like NPs can be divided into two categories: biological extraction and chemical synthesis (Figure 2) and the two methods have their own advantages. Due to the similar polymerization process, melanin-like NPs retain most of properties of natural melanin and have been widely applied in different fields such as oil/water separation, environmental adsorption and so on [19,25]. In recent years, the applications of melanin-like NPs in biomedicine field have achieved much significant results especially in the field of photothermal anti-tumor therapy. In this review, we first introduced the synthesis methods and internal mechanism of melanin-like NPs, and compared the advantages and disadvantages of different methods for reference. Then, we briefly described the inherent physical and chemical properties of melanin-like NPs including photothermal conversion properties, and systematically summarized their applications in nanomedicine during the past decades which mainly divided into two aspects: (1) biological imaging platform. (2) application of melanin-like NPs in PTT. At the end, this review made a comprehensive summary based on the current research status of melanin-like NPs and objectively discussed related technical challenges and obstacles to clinical transformation. This review will help us have a more comprehensive understanding of research status of melanin-like NPs in the field of photothermal therapy, and contributes to promote the further transformation of melanin-like nanomedicine in clinical applications.

## 2. Preparation Method, Polymerization Mechanism and Physical and Chemical Properties of Melanin-Like NPs 

### 2.1. Preparation Method and Polymerization of Melanin-Like NPs

The ways of obtaining melanin-like NPs can be roughly divided into biological extraction and chemical synthesis (Figure 2). Different methods have different polymerization mechanisms. The former method includes direct separation and purification of spherical nanoparticles from cuttlefish ink (others such as black sesame, hair and *Lachnum singerianum* YM296) [26,27,28,29,30], or reprocessing granules of cuttlefish ink to obtain ultrasmall water-soluble melanin nanoparticle [31,32]. Generally, large particles were removed by low-speed centrifugation and then a high-speed centrifugation (18000 rpm, 15 min) were conducted to obtain nanoparticles with a size of approximately 150–200 nm. This physical extraction method was easy to operate but limited by the lack of a unified standard purification technology. Moreover, the composition of these natural nanoparticles lacked of clear composition ratio and chemical structure information. Zhang et al. proved that nanoparticles extracted from cuttlefish ink were mainly composed of melanin, a small amount of amino acids and mono-saccharides [33]. Recently, a new way to obtain bio-derived melanin-like NPs was proposed by Vipul et al. Bacterial outer membrane vesicles (OMVs) containing naturally melanin were obtained through genetically engineered bacteria and extracted by ultracentrifugation [34]. Bacterial genes were easy to modify which was conducive to the later multi- functionalization of melanin-OMVs. In mechanism, these methods all rely on the biosynthesis of melanin in the body. The synthesis of melanin in organisms was a complicated process. According to the presence or absence of sulfur, natural melanin can be divided into eumelanin and pheomelanin, the former does not contain sulfur and the latter contains [21]. Due to the heterogeneity of natural melanin, its precise molecular structure has not been uniformly determined. At present, it is widely recognized that melanin is a biological macromolecule formed from phenolic compounds by oxidative polymerization. After careful chemical research, Henry Stanley Raper established the main process of dopamine oxidation to melanin by tyrosinase and determined that DHI (5,6-dihydroxyindole) and DHICA (5,6-dihydroxyindole-2-carboxylic acid) are the basic components of the eumelanin [35].

**Figure 3:**
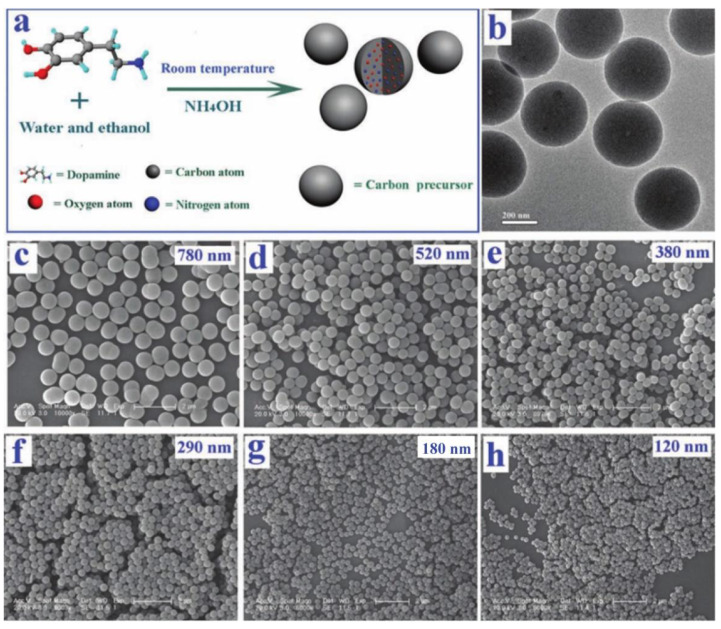
PDA nanoparticles of various sizes. (**a**) Schematic illustration of the synthesis of PDA SMSs. (**b**) Typical TEM image of PDA SMSs with an average diameter of 380 nm. (**c**–**h**) SEM images of PDA SMSs with different diameters prepared at different ratios of ammonia to dopamine [37].

Compared with melanin-like NPs extracted from organisms, artificially synthesized melanin-like NPs have better controllability. Polydopamine nanoparticle (PDA NP) is an excellent representative of synthesized melanin-like nanoparticle and produced by oxidative polymerization of dopamine monomers. There are three methods to synthesis PDA nanoparticles and the most widely used protocol is solution oxidation method [18,36]. This self-polymerization reaction can be realized under mild conditions without complicated equipment. In brief, a certain amount of dopamine hydrochloride was added to the alkaline solution under stirring. Under the action of oxygen, dopamine monomers undergo oxidation reaction and immediately polymerizes, and the color of solution gradually changes from colorless to dark brown. When using this method, the diameter of nanoparticles can be easily controlled through adjusting the amount of dopamine and pH of the solution [37,38,39]. In addition, ethanol could prevent the polymerization of dopamine and the proportion of ethanol in the solution had a certain influence on the dispersion and morphologies of PDA nanoparticles. Lu et al. controlled PDA nanoparticles with granule sizes distribution of 120–780 nm by adjusting the ratio of water, ethanol and ammonia in the mixture solution [40] (Figure 3). Complementary to solution oxidation method, PDA nanoparticles can also be produced by an enzymatic oxidation process. The enzymatic polymerization of precursor molecules such as phenol derivatives and phenolic compounds mimics the synthesis of melanin in organisms to the greatest extent. The related enzymes including tyrosinase, laccase and urease improved the reaction efficiency and the whole polymerization procedure were environmentally friendly [41,42]. This will be a recommended method if the relatively complicated processes of enzyme catalyzed strategy can be satisfied. Alternatively, electropolymerization method is often used to directly deposit PDA nanoparticles on the surface of electrodes. This method is conducive to develop PDA encapsulated nanomaterials and the coating thickness can be controlled by changing a variety of reaction parameters (pH of solution, reaction time and temperature, substrate concentration etc.) However, it is limited that the electropolymerization can be conducted only on the surface of conductive materials [43]. Adjusting the parameters of the synthesis process can also prepare polydopamine nanomaterials of various structures in addition to spherical particles, including nanocapsules, nanofibers, hollow nanocontainers, etc. Therefore, by controlling the synthesis conditions, we can synthesize PDA nanomaterials with different morphologies, sizes and nanostructure.

With continuous improvement of synthesis technology, the existing preparation methods can achieve various types of melanin-like NPs. The polymerization mechanisms of precursor molecules have also been elaborated during the research. In short, the various reaction sites and different degrees of oxidation in the polymerization process generate complex intermediate products, which makes it more difficult to analyze the precise chemical structure of PDA NPs. Fortunately, with the advancement of detection technology, some primary analytical models of PDA NPs have been proposed including covalent bonding mode, etc. [18]. And the accurate chemical structure determination of PDA NPs will be interpreted in the future.

### 2.2. Physical and Chemical Properties of Melanin-Like NPs

#### 2.2.1. UV-Vis Absorption and Photothermal Conversion Performance

Melanin-like NPs have the broadband UV-Vis adsorption spectra that increases monotonically towards the high-energy end [44,45]. This wide range of monotonical absorption profile is hypothesized to be related to disordered primary chemical structures and excitonic effects of secondary structures. The excellent optical properties of melanin-like NPs lie in their ability to efficiently transfer absorbed light energy into heat. Compared to natural melanin, synthetic melanin-like nanomaterials can obtain better optical properties by using computational modeling method to optimize the nanostructures. Due to their distinct properties, melanin-like nanomaterials have gained high attention in the field of photoacoustic imaging and photothermal therapy.

#### 2.2.2. Other Inherent Physicochemical Properties

In addition to outstanding optical properties, other inherent physicochemical properties make melanin-like nanoparticles more attractive. First, they have a strong ability to chelate various metal ions [20,46]. There are various metal coordinate sites on the surface of polydopamine nanoparticles such as amine, semiquinone and phenolic group [47]. And they have been developed as MR contrast platforms for imaging studies in vivo. Melanin in the skin defensed ultraviolet damage by scavenging free radicals produced by radiation. Because of a population of reductive functional groups (e.g., catechol, amine, and imine), melanin-like NPs also exhibit powerful free radical scavenging ability [48]. Liu et al. provided exhaustive activities of melanin-like NPs against multiple RONS including O_2_^·−^, H_2_O_2_, ·OH, etc. [49,50]. Finally, it is worth mentioning that melanin-like NPs show good biocompatibility similar to natural melanin, which greatly improves the safety of applications in vivo.

## 3. Application in Nanomedicine

Melanin-like NPs are superior to many inorganic nanomaterials in the field of nanomedicine due to their own biocompatibility and prominent photothermal properties. And it had been established that PDA NPs with high dose treatment did not show noticeable toxicity in vivo and vitro [24]. Outstanding photothermal conversion ability make melanin-like NPs a more attractive platform for the development of biomedical applications. Here, we focus on their applications in cancer photothermal therapy during the past decade (monotherapy combination therapy and bi-model synergistic therapy) and summarize representative latest research. In addition, this review also introduced other important applications of melanin-like NPs in biomedicine field including biological imaging platform and other diseases treatment (anti-oxidant damage, irradiation protection, etc.).

### 3.1. Biological Imaging Platform

**Figure 4:**
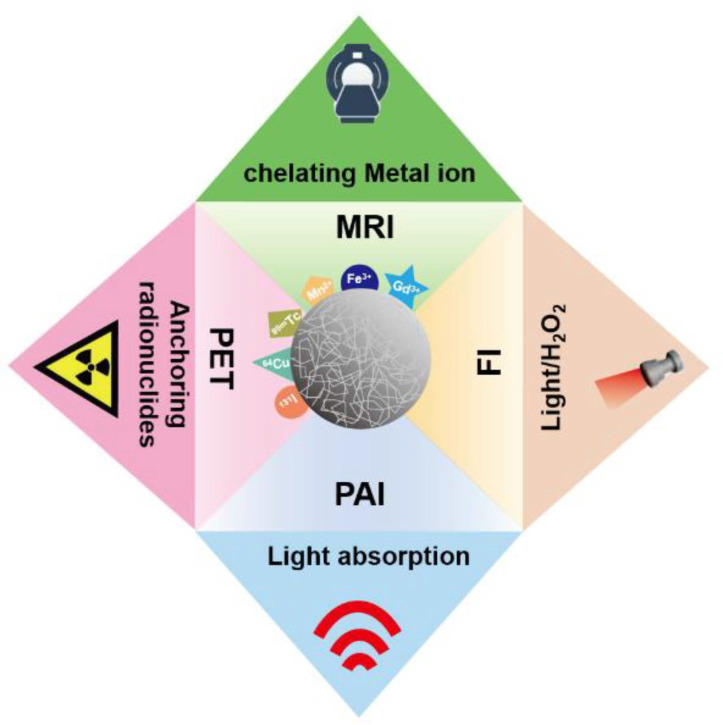
The main bioimaging application of melanin-like NPs.

Due to excellent physicochemical properties, melanin-like NPs have drawn considerable attention in the field of nanoimaging. The inherent photoacoustic imaging (PAI) ability of melanin-like NPs realized high contrast and resolution imaging of tissues which has great application prospects in disease diagnosis and efficacy evaluation. However, some endogenous chromophores such as hemoglobin may cause high background signals [51]. Furthermore, compared with some imaging techniques like PET imaging, PAI shows limited tissue penetration performance. Interestingly, melanin-like NPs can acquire a fluorescent when subjected to severe oxidation conditions and used for intracellular fluorescence imaging (FI). However, FI is limited in intravital imaging due to poor tissue penetration and low sensitivity. Considering the strong metal chelation ability, melanin-like NPs have been exploited for various imaging technologies including magnetic resonance imaging (MRI) and positron emission tomography (PET) to accurately reflect tumor tissue function and metabolism information (Figure 4). In order to obtain complementary diagnostic information, melanin-like NP is also developed for multi-modal imaging in some studies. Representative examples of melanin-like NPs for biological imaging are summarized in Table 1.

#### 3.1.1. Photoacoustic Imaging (PAI)

**Figure 5:**
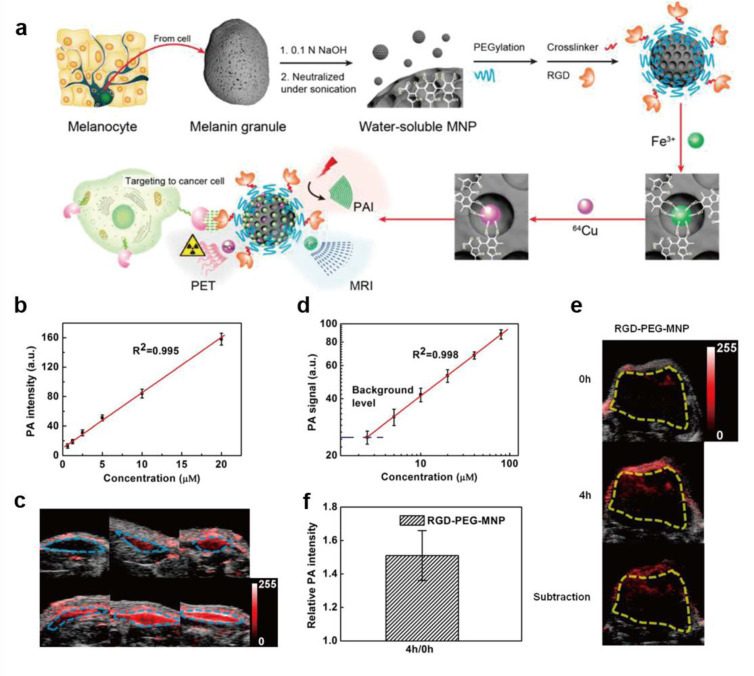
Multimodality molecular imaging of ultrasmall melanin nanoparticles. (**a**) Schematic diagram of synthesis of nanoparticles and multi-model imaging. (**b**) The photoacoustic signal produced by PEG-MNPs at concentrations of 0.625, 1.25, 2.5, 5.0, 10, and 20 μM, and it was observed to be linearly dependent on its concentration (R^2^ = 0.995). (**c**) Photoacoustic detection of PEG-MNP in living mice. (**d**) The photoacoustic signal from each inclusion was calculated. (**e**) The overlaying of ultrasonic (gray) and photoacoustic (red) imaging of U87MG tumor before and after tail-vein injection of RGD-PEG-MNP in living mice. (**f**) Quantitative analysis of enhanced PA signal of U87MG tumor after injection of RGD-PEG-MNP at 4 h [31].

Photoacoustic imaging (PAI) is an image of biological tissues generated based on some endogenous biomolecules radiating acoustic waves under the laser irradiation. The acoustic waves were produced due to tissue chromophores conversed the absorbed light into heat and cause transient thermoelastic expansion [52]. Compared with pure optical imaging technology, PAI exhibits higher spatial resolution and sensitivity in deep tissues which makes PAI a promising technology in the imaging field [53]. Considering the limited optical absorbance of biomolecules in vivo, developing exogenous photoacoustic agents is meaning for clinical applications. Melanin-like NPs have received extensive attention in PAI field due to their inhere biocompatibility and efficient photothermal conversion ability. Fan et al. processed melanin granules to obtain polyethylene glycol (PEG) modified MNPs with high water mono-dispersity and studied the PAI ability of MNPs in vitro and in vivo [31]. The result showed that photoacoustic signal could be detected when the PEG-MNPs concentration was as low as 0.625 μM and it displayed a liner correlation (R^2^ = 0.995) with the MNPs concentrations in the range of 0.625–20 μM (Figure 5). The PAI in living body was also conducted in a U87MG tumor model and a significant photoacoustic signal was detected at 4 hours after injection of RGD-PEF-MNP (200 μM). Similarly, Liopo et al. synthesized PEG-modified melanin-like NPs by oxidative polymerization and evaluated their optoacoustic efficiency in water [54]. The result indicated that the system resolution (<300 μm) and sensitivity (Δμ_α_ = 0.03/cm) in vitro under a safe laser energy density (20 mJ/cm^2^) were both satisfactory. What is more, the optoacoustic efficiency of melanin-like NPs was equal to that of gold nanorods under the equal optical absorption. In addition, Longo et al. prepared melanin granules derives through enzymatic (tyrosinase) oxidation of L-dopa and assessed their potential to be PA agents [51]. It is proved that the PA signal strength increased with an increasing concentration of melanin-like product, from 0.6 to 2.5 mg/mL. Moreover, it could be used as an efficient PA contrast agent in tumor vasculature imaging.

To increase photoacoustic (PA) signal strength in near-infrared window of biological tissue (700–800 nm), Ju et al. proposed a pH induced physical aggregation strategy of melanin-like NPs [55]. He demonstrated that the aggregation of NPs under mildly acidic conditions (i.e., pH 6) caused an increase of PA signal 8.1 times without their absorption tuning. Another way to improve the efficiency of PAI is the enrichment of melanin-like NPs in targeted tissues. About active targeting accumulation, Chen et al. presented poly-L-Lysine (PLL) coated melanin-like NPs which showed strong electrostatic interaction with anionic glycosaminoglycans (GAGs) in cartilage because of the positive charge on the surface [56]. The result demonstrated that the PA signal had a two-fold increase in the normal joint compared to osteoarthritis joint due to the different of GAG content in joint. Recently, Gujrati prepared bacterial outer membrane encapsulated biopolymer-melanin (OMV^Mel^) through genetically engineered Escherichia coli [34]. According to the report, OMV^Mel^ exhibited excellent PA signal response and could accumulate in tumor site through a passive targeting way in a 4T1 mouse mammary gland tumor model. This non-invasive method allows to monitor the spatiotemporal distribution of OMV^Mel^ NPs by using multispectral optoacoustic tomography to detect PA signals. Considering the programmability of E.coli, this bioengineered vesicles are expected to develop into photoacoustic imaging platform for various disease types.

#### 3.1.2. Fluorescence Imaging (FI)

Considering the excellent biocompatibility and optical properties, melanin-like NPs have received extensive attention in the field of fluorescence imaging. Melanin-like NPs exhibit obvious fluorescence property under harsh oxidative conditions (e.g., light exposure and H_2_O_2_) [57,58,59]. In a recent report, Zhang prepared biocompatible fluorescent organic NPs (FONs) through one-pot oxidation of PDA using for cell imaging [60]. This PDA based FONs with tunable photoluminescence showed green and yellow fluorescence under 405 nm and 458 nm excitations, respectively after internalization by NIH-3T3 cells. In another work, Chen et al. obtained F-PDA capsules via polymerization of DA on sacrificial templates in the presence of H_2_O_2_ [61]. The final F-PDA capsules exhibited green influence under the 400 nm laser excitation and the fluorescence intensity was pH dependent (from pH 3–8). After 24 h incubation with HeLa cells, the internalized F-PDAs capsules could be readily visualized with conventional fluorescence microscopy. In addition, there are other kinds of PDA-based FONs developed for fluorescence imaging such as (PEI)-PDA FONs and PDA dots (PDs). Shi et.al reported a polymer-FONs relies on self-polymerization of dopamine and polyethyleneimine (PEI) in rather mild conditions [62]. In vitro characterization showed nanoparticles posse good biocompatibility and could be potential used for biomedical application. In addition, two-photon excitation fluorescence of melanin has been widely reported. Two-photon absorption (TPA) imaging of melanin showed higher sensitivity and micrometer resolution [63,64,65]. Lai et al. proposed a stepwise multiphoton activated fluorescence (SMPAF) of melanin which activated by a continuous-wave mode NIR laser. The result showed that SMPAF imaging could not only locate melanin reliably but also eliminate background interference from other components inside mouse hair and skin [66]. 

**Table 1:** Examples of melanin-like NPs for bioimaging applications.

Melanin-Like NPs Type	Size (nm)	Model	Modality	Reference
MNPs	<10 nm	U87MG/HT29 tumor	PAI/PET/MRI	[28]
^89^Zr-MMPP	4.5 nm	AKI mice	MRI/PET	[29]
OMV^Mel^	20–100 nm	4T1	PAI	[31]
Melanin-free acid	6.9 ± 1.2 nm	Breast cancer	PAI	[47]
PDA	48 ± 12 nm	mice	PAI	[50]
PDA	130 nm	B16 melanoma	PAI	[51]
MNPs	40 nm	Osteoarthritis	PAI	[52]
PDA-FONs	A broad scale	HIN-3T3 cells	FI	[56]
F-PDA	60 ± 10 nm	Hela cells	FI	[57]
PDA	~50 nm	A549 cells	FI	[58]
Fe^3+^-MelNPs	98/318/570 nm	mice	MRI	[67]
Fe^3+^,^64^Cu^2+^-MNPs	16.4 nm	HT29	PET/MRI/PAI	[68]
Mn^2+^-MNP	~5.6 nm	HeP-2 tumor	MRI	[69]
MnEMNPs	40–150 nm	U87MG	PAI/MRI	[70]
Gd^3+^,^64^Cu^2+^-MNPs	13 nm	Nude mice	MRI/PET/PAI	[71]
^64^Cu^2+^-MNPs	~60 nm	HepG2 tumor	PET/PAI	[72]
MNP-Ag-^131^I	12 nm	PC3 tumor	SPECT	[73]

#### 3.1.3. Magnetic Resonance Imaging (MRI)

MRI is widely used in clinical disease diagnosis due to the superb spatial resolution and can provide clear images of soft tissue structure. However, its major drawback is low sensitivity inherent which promotes the development of contrast agents. Generally speaking, MRI contrast agents can be divided into longitudinal relaxation contrast agents (*T*_1_) and lateral relaxation contrast agents (*T*_2_) according to different mechanisms [67,68]. Among them, Gd^3+^ contrast agents are the representative of *T*_1_-agents and have been applied in the clinical diagnosis. However, non-specific distribution and rapid metabolism in vivo make them difficult to enrich in the lesion site [74]. Moreover, Free Gd ions in vivo may cause severe toxicity [69]. For example, a study showed that patients with renal dysfunction treated with Gd-based contrast agents may induce nephrogenic systemic fibrosis [70]. The application of *T*_2_ contrast agents is limited because *T*_2_ signal is easily interfered. In addition, it is reported that superparamagnetic Fe_3_O_4_ (a common *T*_2_ contrast agent) has potential toxicity in vivo [68]. Nanoparticles appear attractive advantages in circulation in the body and targeted drug delivery and melanin-like NPs have been a preferable candidate for enhanced MRI.

**Figure 6:**
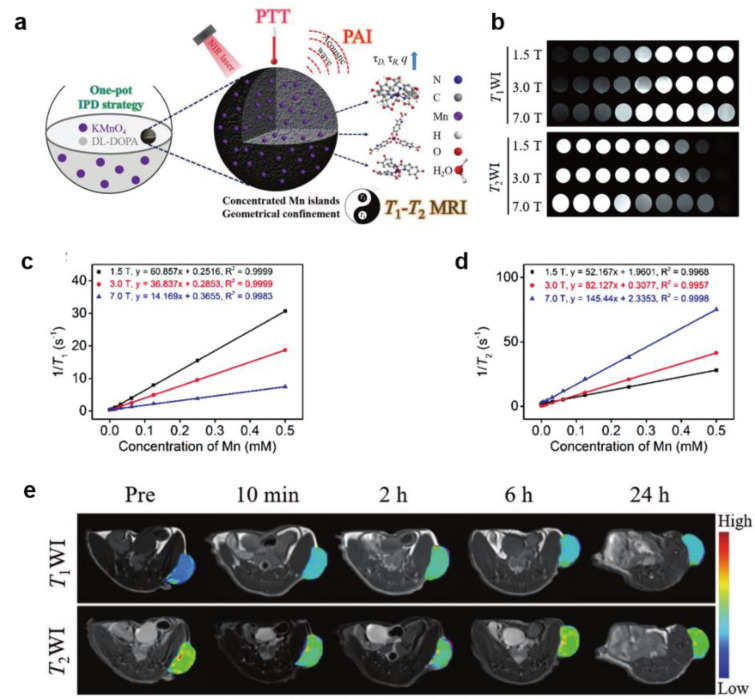
Manganese doped melanin-like nanoparticles for dual-model MRI in vitro and vivo. (**a**) Synthesis procedure and theranostic applications of Mn^2+^-melanin-like NPs. (**b**) *T*_1_ WI and *T*_2_ WI at various magnetic fields (MFs). The linear relationship for the (**c**) *r*_1_ and (**d**) *r*_2_ relaxivities of MnEMNPs as a function of Mn^2+^ concentration at various MFs. (**e**) In vivo theranostic evaluation of U87MG tumor as a function of time after injection with MnEMNPs [79].

At present, melanin-like NPs have been employed for chelating various paramagnetic metal ions including Gd^3+^, Fe^3+^, Mn^2+^, Cu^2+^ etc. as MR contrast agents [71,72,73,75]. For example, Ju et al. reported a complementary material of bio-inspired Fe^3+^-melanin-like NPs with significant enhancement to T1-weighted MRI contrast (17 mM^−1^s^−1^ at 3.0 T) [76]. In addition, increasing the aggregation of nanoparticles at the tumor site can achieve clearer tissue imaging by controlling the particle size (<10 nm) or modifying the targeted molecules. Recently, Yang synthesized ultrasmall melanin-like NPs chelating Fe^3+^ ions and then embedded them into the cavity of apoferritin (APF) to target transferrin receptor 1 (TfR1) [73]. The final nanoparticles improved MRI sensitivity in HT29 tumor model attributed to targeting property combined with the enhanced permeability and retention (EPR) effect. Given that Mn^2+^ ion is one of the trace elements for metabolism of human body, manganese doped melanin-like NPs were also developed for enhanced MRI imaging [77]. For example, Xu synthesized ultrasmall melanin NPs chelating Mn^2+^ (MNP-PEG-Mn) which possess much higher *r*_1_ longitude relaxivity (20.56 mM^−1^s^−1^ at 3.0 T) than that of Gadodiamide (6.00 mM^−1^s^−1^) [78]. What is more, this kind of nanoparticles could be efficiently cleared by renal and hepatobiliary pathways and showed negligible toxicity to organs. Similarly, Liu et al. prepared a kind of novel engineered manganese-eumelanin coordination nanocomposites by a facile one-pot intra-polymerization doping approach. This Mn^2+^-melanin-like NPs exhibit ultrahigh longitudinal relaxivity (*r*_1_ and *r*_2_) attributed to the high manganese doping efficiency (> 10%) and geometrically confined conformation [79]. Compared to single imaging, this high-performance longitudinal-transverse (*T*_1_–*T*_2_) dual-model magnetic imaging of Mn^2+^-melanin-like NPs can provide complementary image information and promote the development of multifunctional imaging nanoplatforms (Figure 6).

#### 3.1.4. Radionuclide Imaging

**Figure 7:**
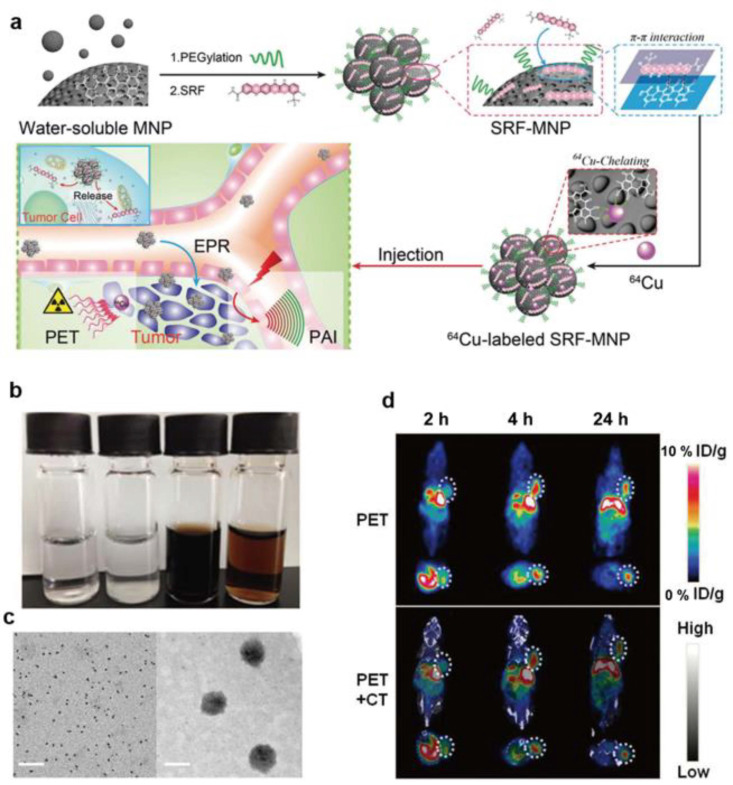
The preparation of ^64^Cu^2+^ chelated SRF-MNPs and in vivo PET images of nanoparticles. (**a**) Schematic illustration of the preparation of SRP-MNPs and therapy process in vivo. (**b**) The photographs of different groups (from left to right): (1) pure water, (2) SRF precipitated in PBS, (3) PEG-MNP dispersed in PBS, and (4) SRF dispersed in PBS having PEG-MNP. (**c**) TEM of PEG-MNP (left) and SRF-MNP (right), scale bar = 50 nm. (**d**) Representative decay-corrected coronal (top) and transaxial (bottom) small-animal PET images and overlaid CT and PET images of HepG2 tumor (region enveloped by white dotted line) acquired at different times post injection with ^64^Cu^2+^ chelated SRF-MNPs [82].

Radionuclide imaging mainly includes positron emission tomography (PET) and single-photon emission computed tomography (SPECT) imaging. In principle, they rely on the detection of high-energy rays released when radionuclides decay in the body and posse the advantages of high sensitivity and precise quantification [80]. Nowadays, the use of melanin-like NPs combined with various radionuclides (such as ^99m^Tc, ^64^Cu^2+^, ^131^I, and ^89^Zr) for PET imaging has received a lot of attention. By functional modification, melanin-like NPs can give the loaded radionuclide with targeting ligand properties and alter their pharmacokinetics in vivo. There are many related reports conforming this, for example, Hong et al. prepared radioactive ^64^Cu^2+^ labelled Melanin-like NPs with the 85% radiolabeling yield. The result showed ^64^Cu^2+^-MNP can efficiently accumulate in mouse liver and can be used for PET imaging [81]. In another study, ^64^Cu^2+^ was chelated to MNP-SRF nanoparticles to prepare a water- soluble nanocomplex for PEI imaging and tumor therapy [82]. The result showed that ^64^Cu^2+^ were successfully labeled the NPs with a yield of 64% via simple mixing of them. To further monitor the temporal and spatial distribution of NPs in vivo, HepG2 tumor bearing mice were administrated with complex NPs and detected the radioactivity in tumor sites at different times. The result showed that ^64^Cu^2+^ radioactivity in tumors were significant increased at 4 h after injection and then gradually decays over time (Figure 7). Given that melanin-like NPs are easy to functionally modify, Fan prepared RGD modified ^64^Cu^2+^-PEG-MNPs with about 80% radiolabeling yield [31]. A U87MG tumor bearing mice model was used to detect the distribution of NPs in vivo. As expected, PET signal in tumor site of ^64^Cu^2+^-PEG-MNPs increased within 24 h after administration of nanoparticles and finally reached 5.93 ± 0.89% ID/g at 24 h. Similar results had also been observed in the HT29 human colorectal cancer model overexpressing TfR1. In another work, Sun chelated ^89^Zr to manganese-doped melanin-like NPs (^89^Zr-MMPP) for PET imaging and treatment assessment in acute kidney injury (AKI) mice [32]. In addition, ^131^I and ^99m^Tc are also used to label melanin-like NPs for PET imaging to assist disease detection or treatment [83,84].

### 3.2. Application of Melanin-like NPs in PTT

Compared with traditional radiotherapy and chemotherapy, PTT has characteristic advantages in cancer treatment, such as precise controllability of the irradiation site and intensity, better specificity and lower systemic side effects. Considering these outstanding superiorities, PTT has received widespread attention since its proposal [4,6]. Currently, various photothermal conversion agents (PTCAs) with strong NIR absorption including organic and inorganic materials have been developed for tumor photothermal ablation (Figure 1). Despite appreciable success, the long-term safety and effective half-life of existing PTCAs in vivo limit their clinical application. Therefore, excellent biocompatibility and high photothermal efficiency are essential properties of PTCA for clinical applications. Melanin-like NPs are fully meet these two clinical application factors. It is reported that their photothermal conversion efficiency can reach to 40%, higher than that of many reported PTCAs [20,85]. In addition, the abundant groups on the surface of melanin-like NPs contribute to the further functional modifications for enhanced imaging or treatment. Base on the above excellent characteristics, melanin-like NPs have become preferred candidates for clinical PTT. So far, cancer photothermal therapy research based on melanin-like NPs is mainly divided into two aspects: (1) imaging-guided PTT monotherapy, (2) combination therapy strategies including PTT. Examples of melanin-like NPs for tumor PTT are summarized in Table 2.

#### 3.2.1. Imaging-guided PTT Monotherapy

**Figure 8:**
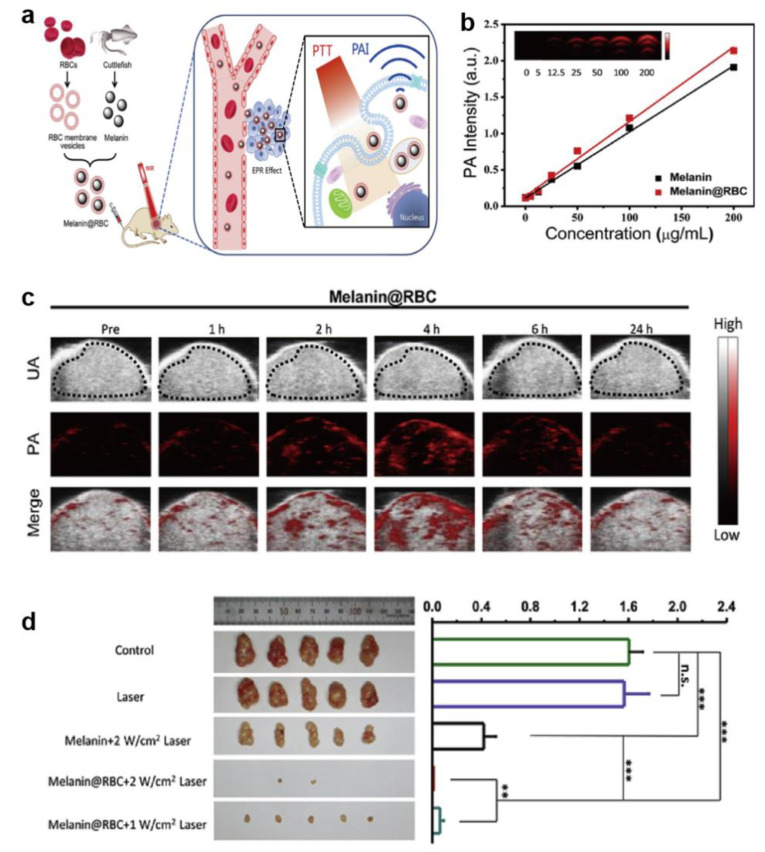
Melanin @ RBC NPs for enhanced PTT. (**a**) Schematic illustration of the preparation of red blood membrane camouflaged melanin nanoparticles and PAI guided PTT. (**b**) PA signal versus various concentrations of melanin or Melanin @ RBC NPs in vitro. Inset is the PA images of Melanin @ RBC NPs with different concentrations. (**c**) Ultrasound (UA) images, PA images, and merged images in tumor region before and after intravenous injection of Melanin @ RBC NPs in A549 tumor-bearing mice. (**d**) Photos of tumors dissected from each group on the 13th day after PTT and comparison of tumor weight of different groups [26]. (** *p* < 0.01, *** *p* < 0.001 and n.s. represents no significance.)

Photothermal therapy has become a promising treatment since it was proposed. Photothermal ablation based on nanomaterials has attracted more and more attention due to its controllability in vivo and in vitro. Nevertheless, many previously reported photothermal conversion agents have different defects such as low photothermal conversion efficiency and potential tissue toxicity. Therefore, the development of new photothermal conversion agents is of great significance for cancer PTT [6,86]. Compared with traditional PTCAs such as organic small molecule and inorganic nanomaterials, Melanin-like NPs are highly superior for in vivo photothermal therapy, mainly in the following aspects: good biocompatibility, negligible long-term tissue toxicity and high photothermal conversion efficiency [20]. In the study of Kim et al., a thermo hydrogel containing melanin was prepared for anti-tumor therapy in mouse CT26 models. The result showed that with 808 nm NIR laser irradiation, the temperature of tumor tissue increased to 55 °C after intratumoral injection of Pol-Mel, much higher than that of control group (38 °C). In vivo anti-tumor evaluation result indicated that the photothermal effect alone of melanin hydrogel could inhibit tumor growth effectively [87]. Considering the natural advantages of melanin-like NPs in biological imaging, imaging-guided photothermal anti-tumor therapy has been widely explored. PAI or MRI based on melanin-like NPs can provide the temporal and spatial distribution of NPs in organisms and it can be used for effectiveness evaluation to assist anti-tumor therapy. Liu et al. synthesized Dpa-melanin NPs with an average diameter of approximately 160 nm by oxidation and self-polymerization and the photothermal conversion efficiency could reach 40% which was much higher than that of Au nanorods (22%) [24]. The final NPs were injected intratumorally into Balb/c mice bearing 4T1 tumors to assess their anti-tumor potential in vivo. The result showed that after the NIR irradiation, tumors in treatment animals were ablated without regrowth or with rather slow growth compared with that of control group. Further, they fabricated Gd-DTPA-modified Dpa-melanin CNSs for T1-weight MRI imaging in tumor site to investigate their application in MRI and therapy of tumor in vivo. In another research, bio-inert silica was coated on the surfaces of Gd-chelated melanin NPs (Gd-Mel@SiO_2_ NP) to obtain enhanced dual-model contrast for PTT [88]. The SiO_2_ nanocoating caused a significant brighter MR *T*_1_ contrast effect comparing with control groups due to increased out sphere water diffusion time. What’s more, the SiO_2_ nanocoating provided protection for labelled fluorescent molecules from being quenched by melanin to improve the fluorescence intensity. Finally, the enhanced dual-modal imaging guided catheter-directed infusion PTT showed significant tumor growth suppression effect in clinically relevant human prostate cancer xenograft model. Inspired by Gd^3+^-chelated melanin-like NPs, Mn^2+^ is also employed to combined with melanin-like NPs for MRI-guided photothermal therapy. For example, Miao et al. successfully prepared PEGylated Mn^2+^-chelated polydopamine (PMPDA) NPs for MRI guided PTT. The PMPDA NPs exhibited distinct MRI signal enhancement for both in vitro and in vivo imaging [77]. The longitudinal relaxivity coefficient (*r*_1_) of PMPDA NPs was 6.55 mM^−1^s^−1^ at 9.4 T, much higher than that of Gd-DTPA (a commercially available contrast agent). In addition, this NPs can effectively kill Hela cells due to their photothermal property under the laser irradiation and can be a promising platform for MRI guided PTT of cancer cells. In another meaningful report, Liu and his collaborators proposed a kind of novel engineered manganese-eumelanin coordination nanocomposites (MnEMNPs) which can achieve high-performance longitudinal-transverse (*T*_1_–*T*_2_) dual-model MRI/PAI and photothermal tumor ablation [79]. In the research of Lin, ^64^Cu was coupled with magnetic melanin nanoparticles (^64^Cu-MMNs) for PET/MR/PA multi-model imaging guided tumor PTT and protection from UV and γ-irradiation [75]. Given that natural melanin NPs extracted from organisms possess many advantages such as environmental friendliness and easy preparation, Chu et al. obtained sheet-like structure black sesame melanin (BSM) by extracting from black sesame seeds. This BSM showed potential for sentinel lymph node (SLN) mapping and could be used for cancer PTT. The result showed that liposome-BSM significantly suppressed Eca-109 esophagus carcinoma growth after liposome-BSM treatment [28]. In order to improve the blood circulation time of melanin-like NPs and prevent them from being eliminated by the immune system, a feasible strategy is proposed: camouflaging nanoparticles with bio-membranes [89,90,91]. Yang’s team reported a red blood cell membrane- coated melanin (Melanin @ RBC) NPs as a platform for PAI and PTT in vivo [26]. Results showed that at 24 h after intravenous injection, the blood retention of Melanin NPs was decreased to 1.09 ± 1.35% ID/mL while about 11.16 ± 2.82% ID/mL Melanin @ RBC NPs still in blood circulation. The PA signal at tumor site peaked at 4 h post injection and the average intensity of Melanin @ RBC group was higher than that of Melanin group at the time. Further, A549 tumor-bearing mice were used to investigate the antitumor efficacy and the Melanin @ RBC NPs showed better tumor inhibition (almost 100%) compared with Melanin NPs (about 78%) (Figure 8). Yang’s team further prepared RBC membrane and MCF-7 cell membrane hybrid membrane (RBC-M) camouflaged melanin NPs (Melanin @ RBC-M) for enhancing efficacy of PTT. As expect, the final NPs showed prolonged blood circulation and homotypic targeting to source MCF-7 cells simultaneously. This hybrid membrane coating melanin NPs exhibited excellent PAI in vivo and successfully suppress tumors growth in MCF-7 tumor-bearing nude mice [27]. 

#### 3.2.2. Combination Therapy Strategies Including PTT

Tumor PTT shows alluring advantages compared to traditional therapy, especially that it can be controlled to minimize the damage to non-targeted tissues. However, PTT alone is difficult to completely ablate large tumors due to uneven temperature distribution of tumor site. It needs to a quick rise in local cellular temperature above the threshold value of 43–45 ℃ and continue for a period of time (15–60 min) to kill cancer cells. Inevitably, some marginal cancer cells survived because of insufficient heat intensity. Surviving border cells in tumor tissue further promote cancer recurrence and metastasis, which greatly reduces the anti-tumor efficiency [5,86]. To solve this problem, combination therapy strategies is proposed for enhanced overall efficacy.

**Figure 9:**
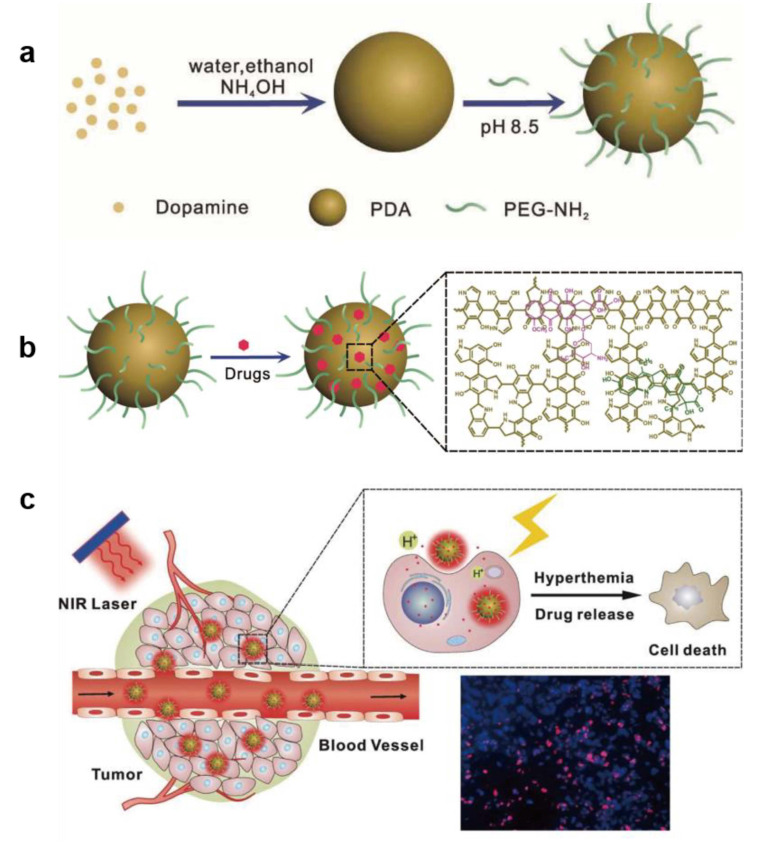
The synthesis (**a**), drug loading behavior of PDA-PEG (**b**) and scheme presentation of PTT-CT synergetic treatment of tumor (**c**) [94].

Compared to nanomaterials, small molecule chemotherapeutics have deeper penetration in tumor tissues but most of them face the dilemma of poor water solubility and short-term blood circulation time. Therefore, melanin-like NPs loading drug molecules can effectively combine the advantages of PTT and chemotherapy [92,93]. After targeted delivery to tumor tissue, photothermal-chemotherapy (PT-CT) eliminates most tumor cells, showing robust anti-tumor responses. For example, doxorubicin (DOX) and 7-ethyl-10-hydroxycamptothecin (SN38) were loaded on PEG-PDA NPs by π–π stacking and/or hydrogen binding [94]. The drug-loaded NPs showed great stability and drug-retaining capability under physiological conditions and the drug release from final NPs with different stimuli (NIR light, pH and reactive oxygen species) was analyzed. To investigate the synergetic effect of PTT and CT, PC-9 tumor-bearing mice were injected with different recipes with or without NIR irradiation. The therapeutic studies demonstrated that synergetic therapy group was significant suppress tumor growth compared with PTT alone or chemotherapy alone (Figure 9). Similarly, Gao reported PDA loading DOX for synergistic therapy of cancer cells [95]. In another work, indocyanine green (ICG) was loaded simultaneously to achieve red-shifted NIR absorbance of final NPs and enhance photostability. The results showed that enhanced PTT and CT combined therapy achieved a remarkable effect compared with respective single treatment modality [96]. In Li’s work, arginine-glycine-aspartic-cysteine (RGDC) peptide was successfully modified on the PDA surface (PDA-RGDC) to target enrichment of nanoparticles at the tumor site. Chemotherapy drug DOX was next loaded on PDA-RGDC which could be released by NIR light and pH dual-stimuli. The results of treatment experiments showed that PTT-CT synergistic therapy could efficiently eliminate tumors [97].

**Table 2:** Examples of melanin-like NPs for photothermal therapy (PTT).

Melanin-Like NPs Type	Model	Application	Reference
Melanin @ RBC	A549	PAI-guided PTT	[23]
Melanin @ RBC-M	MCF-7	PAI-guided PTT	[24]
Liposome-BSM	Eca-109	PTT	[25]
CINPs	CT26	PTT-immunotherapy	[30]
Dpa-melanin CNSs	4T1	MRI-guided PTT	[46]
^64^Cu^2+^-MMNPs	U87MG	Multimodal-guided PTT	[64]
MnEMNPs	U87MG	PAI/MRI-guided PTT	[70]
Mn^2+^-PDA	4T1	MRI-guided PTT	[74]
Pol-Mel	CT26	PTT	[78]
Gd-Mel@SiO_2_ NPs	PC3	MRI/FI-guided PTT	[79]
Melanin-based liposome	BxPC-3	CT-PTT	[83]
PDA-PEG	PC-9	CT-PTT	[84]
PDA-ICG-PEG/DOX(Mn)	4T1	CT-PTT	[86]
PDA-RGDC	Hela	CT-PTT	[87]
PDA/mCaP H-JNPs	HepG-2	CT-PTT	[89]
^64^Cu-PDA	U87MG	CT-PTT	[90]
Melanin-dot	U87GM	CT-PTT	[91]
Melanin-based vaccine patch	B16F10	PTT -immunotherapy	[98]
Fe@PDA-PEG	CT26/4T1	PTT-immunotherapy	[99]
PHPD-NPs	MDA-MB-231	PDT-PTT	[100]
PDA-Ce6	HepG2	PDT-PTT	[101]

**Figure 10:**
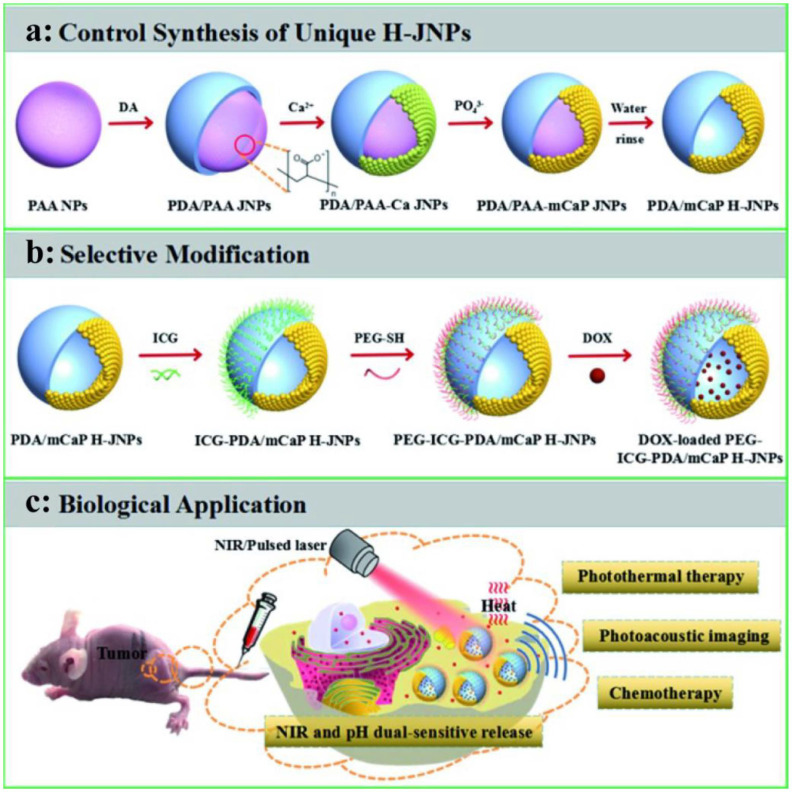
The controlled synthesis of PEG-ICG-PDA/mCaP H-JNPs (**a**) and biofunctionalization of multifunctional H-JNPs (**b**). (**c**) PA imaging-guided chemo-photothermal synergistic cancer therapy [98].

Compared with the traditional PDA NPs, dopamine-coated nanoparticles are expected to achieve advantages integration of the two materials [5,102]. Nam developed polydopamine-coated spiky gold NPs as a new photothermal agent combining with DOX for high-efficiency anti-tumor treatment. In a CT26 colon carcinoma mice model, 85% of mice showed satisfactory treatment effects on local as well as distant tumors. Interestingly, Zhang obtained spherical polydopamine/mesoporous calcium phosphate hollow Janus NPs (PDA/mCaP H-JNPs) through a novel and facile approach [98]. ICG and DOX were loaded to the precise synthesis NPs to achieve superior PA imaging capability and improved anti-cancer effect. Moreover, methoxy-poly (ethylene glycol) thiol (PEG-SH) were modified on PDA domains to ensure the stability of NPs in physiological conditions (Figure 10). As expected, such multifunctional NPs showed powerful anti-tumor capability with chemo-phototherapy combined strategy. Recently, melanin-like NPs encapsulating various metal ions for imaging-guided anti-tumor therapy have also been extensively studied. Herein, radionuclide-64Cu was loaded on PDA-gadolinium-metallofullerene core to obtain satellite nanotheranostic agent (CDPGM) which posse multi-model imaging ability (PET, MRI and PAI). What is more, this CDPGM NPs were further loaded with DOX to achieve multimodal imaging guided chemo-phototherapy combined anti-tumor effect [99]. In addition, Sun proposed a dual-model imaging nano-agent that incorporates discrete Pt (II) metallacycle and fluorescent into multifunctional melanin dots. PAI and NIR-II imaging showed that nanoparticles accumulated at tumor sites. The nano-agent exhibited a superior antitumor performance and less side effects than a single treatment due to the high efficiency of chemo-photothermal synergistic therapy [103].

**Figure 11:**
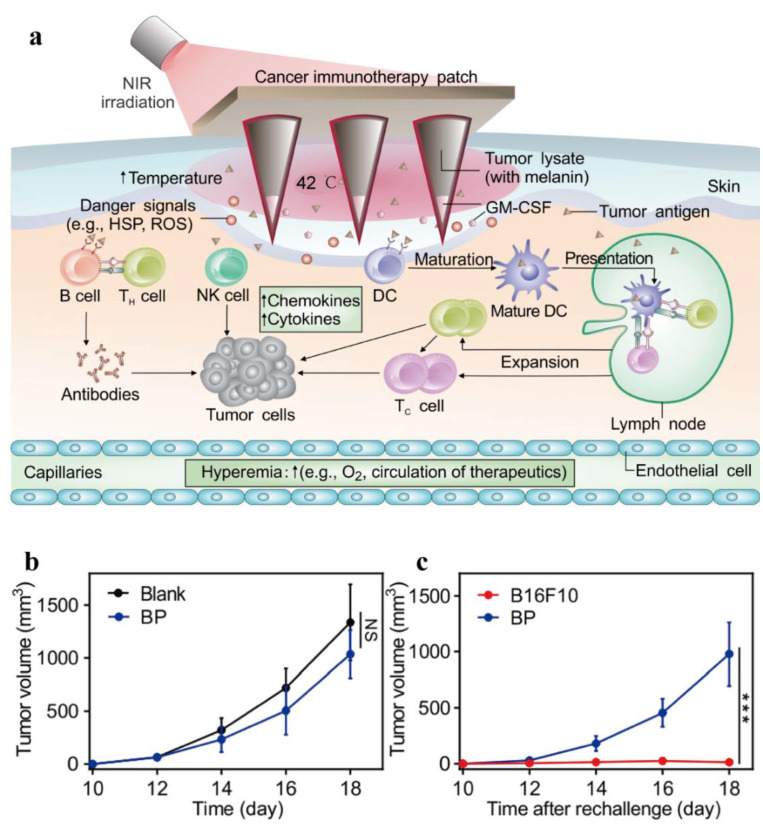
A transdermal MN-based vaccine patch for photothermal assisted immunotherapy. (**a**) Schematic illustration of melanin-based transdermal vaccination. (**b**,**c**) anti-tumor effect of local cancer immunotherapy treatment toward distant B16F10 tumors. Average tumor volumes in vaccinated mice rechallenged with either B16F10 cells or BP cells on day 80 (**b**) and Kaplan-Meier survival curves for rechallenged mice (**c**) [109]. Data points represent mean ± SD (*n* = 8). Error bars indicate SD. Statistical significance was calculated by Student’s *t* test and log-rank test (NS, *p* > 0.05; *** *p* < 0.001).

The immunosuppressive microenvironment plays an important role in tumor occurrence and development. Immunotherapy has been an attractive antitumor strategy and exhibits unique superiority including great specificity and long-term efficacy. [100,104,105]. However, immunotherapy alone has limited antitumor efficacy in vivo due to various reasons. For example, Immune checkpoint inhibitors (PD1/PD-L1, CTLA-4) have achieved certain success in cancer treatment by block the immune evasion of cancer cells but they are limited in clinical applications mainly due to large individual variations and low therapeutic responses (about 20%) [101,106]. In addition, possible toxicities and inappropriate immune responses, such as cytokine‑release syndrome (CRS) have also caused attention [107,108]. Recent studies have shown that photothermal ablation causes cancer cell necrosis, releasing tumor related antigens and pro-inflammatory cytokines which may activate immune response to suppress not only primary tumors but also distant metastatic tumors [86]. Therefore, PTT combined with immunotherapy has become a promising anti-tumor strategy. Ye proposed a B16F10 melanoma vaccine patch which can target antigen-presenting cells (APCs) directly to promote immune activation [109]. In this design, the whole tumor lysate of B16F10 as a broad source of tumor-associated antigens elicit powerful immune responses and the presence of natural biological material melanin in the lysate promoted tumor-antigen uptake by dendritic cells by locally controlling temperature rise (42 ℃). The mild increase of local temperature contributed to increase blood perfusion and promote lymphatic circulation that further facilitates the migration of APCs and T cell to enhance specific immune response. It is worth mentioning that intradermal microneedle (MN) achieved gradual release of tumor antigens after inserting into the skin and further facilitated the uptake and presentation of antigens by DCs, which is beneficial to the immune activation of lymphatic vessels in the dermis. As expected, this B16F10 melanoma vaccine patch showed power antitumor effect both in primary tumor and distant tumor in B16F10 melanoma-bearing mice. In addition, *BRAF^V600E^*-mutated BP melanoma model and triple-negative breast cancer 4T1 carcinoma model was also used to demonstrate the potency of proposed vaccination. Similar tumor regression and long-term survival were obtained in these two modes. 75% and 87% of mice resistant to BP and 4T1 engraftment respectively were achieved by this combined therapy approach (Figure 11).

**Figure 12:**
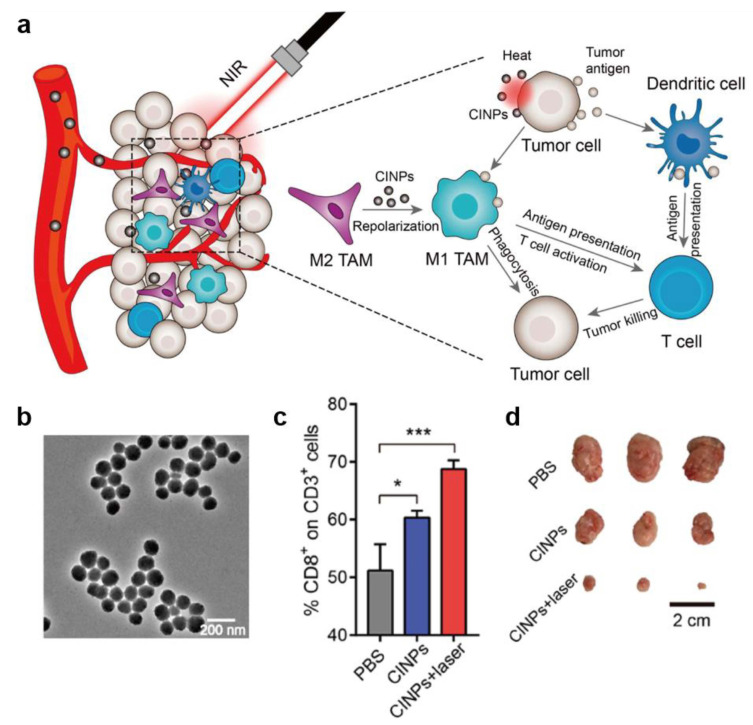
Nanoparticles from cuttlefish ink for inhibiting tumor growth by inducing macrophage repolarization and synergizing photothermal therapy. (**a**) Schematic illustration of anti-tumor mechanism of CINPs in vivo. (**b**) TEM images of CINPs. (**c**) Relative quantification of CD8^+^ T cells in CD3^+^ T cells within tumors following different treatments. (**d**) Representative tumor images of different groups after 16 days’ treatment [33]. Data are presented as the mean ± SD (*n* ≥ 3). * *p* < 0.05, *** *p* < 0.001.

In another research, a melanin-like nanoparticles extracted from cuttlefish ink (CINPs) were employed for tumor growth inhibition by synergized immunotherapy and PTT [33]. According to the report, CINPs consisted of multiple components including melanin, polysaccharides, oligopeptides, metals, etc. and could repolarize M2 TAMs toward antitumor M1 phenotype through the activation of mitogen-activated protein kinase (MAPK) signaling pathway. Considering the excellent photothermal conversion ability of melanin, a reasonable anti-tumor strategy was proposed by inducting macrophage repolarization and synergizing PTT. On the one hand, repolarization of M2 macrophage into M1 phenotype caused effective anti-tumor effect through phagocytosis of tumor cells and production of various antitumor factors including TNF-α, IL6, etc. On the other hand, the photothermal effect of melanin generated tumor-associated antigens which elicited an effective T cells response. In vivo experiment, this natural melanin-like NPs showed potent tumor suppressor effect and almost completely inhibited the tumor growth in CT26 tumor bearing mice (Figure 12).

**Figure 13:**
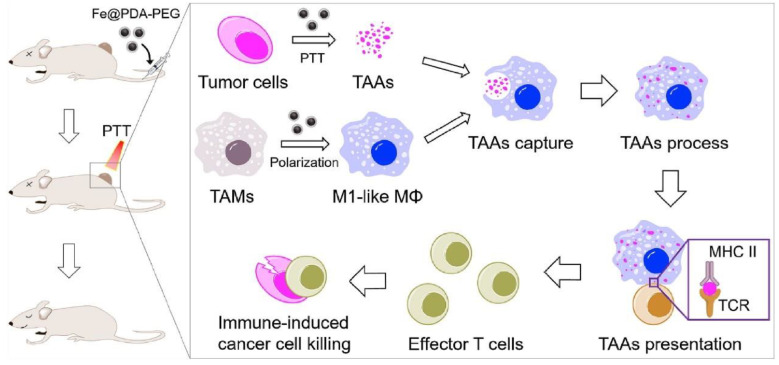
Fe@PDA-PEG induced M2-like TAMs to M1 combined with PTT-induced tumor-associated antigens release for combined anti-tumor therapy [111].

In addition to natural melanin-like NPs, artificially synthesized PDA NPs can also re-polarize M2 TAM into M1 phenotype by chelating Fe^3+^. Recently, Rong et al, achieved synergistic anti-tumor therapy of PTT and immunotherapy by preparing iron chelated PEG-PDA NPs (Fe@PDA-PEG) [110]. Compared with elimination of M2 TAM, repolarization them into M1 anti-tumor phenotype is more attractive which can not only mitigate the TAMs immunosuppression bust also evoke antitumor responses [111,112]. Here, iron chelation could repolarization TAM into M1 mode and PDA NPs were used for photothermal ablation of tumor cells. The result showed that Fe@PDA-PEG NPs could increase the ratio of M1 macrophage compared with control group in vitro and in vivo. Moreover, this melanin-like NPs showed antitumor efficacy on both subcutaneous colon tumor and orthotopic breast cancer (Figure 13).

There are variety combined treatment strategies were developed based on photothermal therapy of melanin-like NPs. In addition to the above-mentioned synergetic treatment approaches, photodynamic therapy (PDT) and PTT combination has also received extensive attention [113]. Melanin-like NPs such as PDA can improve the stability and metabolism of small molecule photosensitizers in vivo for effective tumor therapy. For example, Han proposed a PDA nanoparticles coating photosensitizer-conjugated hyaluronic acid for targeted photo-mediated tumor therapy [114]. In this design, PS-HA shell was used for cancer targeting while PDA core played role as a quencher for PSs. This synthesized PS-HA-shielded PD-NPs (PHPD-NPs) released photosensitizers in tumor site by degrading hyaluronic acid (response to hyaluronidase) and exhibited a combined anti-tumor effect of PDT and PTT. Recently, PDA NPs loading chlorin e6 (Ce6) with the polymer matrix was developed for combined therapy of PDT and PTT [115]. Under the 665 nm laser irradiation, the nanoparticles exhibited the combined killing to bladder cancer cells through photodynamic from Ce6 and photothermal from PDA. It is worth mentioning that PDA NPs as a multifunctional carrier platform for monotherapy and other combination therapy have also been extensively reported [116,117,118]. However, they are beyond the scope of PTT of melanin-like NPs and not be described in detail.

### 3.3. Application of Other Properties of Melanin-Like NPs

Melanin-like NPs play an important role in the treatment of various disease except cancer due to their excellent physical and chemical properties and the following is a brief introduction of some representative applications. Firstly, melanin-like NPs enrich reductive functional groups such catechol and imine which can effectively scavenge multiple reactive oxide species (ROS) in vitro and in vivo [20,48]. Therefore, they are widely used for various disease related to ROS damage such as brain injury in ischemic stroke [50], acute inflammation-induced injury [49], acute kidney injury [32] and periodontal [119]. In addition, melanin-like NPs such as PDA nanoparticles posse excellent adhesive property and metal chelating ability which can be used for antibacterial agents [120,121,122,123]. What’s more, PDA NPs can serve as dopamine replenisher or drug carrier to improve neuroprotective efficiency in Parkinson’s disease (PD) [124,125]. Finally, melanin-like NPs are also involved in other biological applications such as wounding healing and irradiation protection which is a promising multifunctional biological material.

## 4. Conclusions and Perspectives

In this review, the synthesis methods, internal mechanism and physicochemical properties of melanin-like NPs were described systematically. At the same time, it also comprehensively summarizes the applications of melanin-like NPs in photothermal therapy for cancer during the past ten years. This excellent photothermal conversion agent showed powerful antitumor effect no matter in single photothermal therapy or combination therapy. What is more attractive is that they possess good biocompatibility and safety due to the similar chemical structure to natural melanin. Accordingly, melanin-like NPs are expected to become a new generation candidate for clinical photothermal therapy. We believe that this featured article will contribute to understand the latest research trends of melanin-like NPs in the field of photothermal therapy and has certain reference value in subsequent research. Despite appreciable success has been achieve of PTT based on melanin-like NPs, there are still some technical limitations and scientific issues to be solved before clinical translation.

The current ways to obtain melanin-like NPs mainly include biological extraction and chemical synthesis which can meet the small-scale needs of scientific research and there is still much room for exploration in industrial production. A controllable, non-polluting, low-cost mass manufacturing technique needs to be developed. In addition, the propose of novel melanin-like NPs synthesis method such as controllable biosynthesis in vivo can achieve the integration of preparation and delivery, which is of great significance for breaking the limitations of present approaches. When it comes to polymerization mechanism, a more perfect measurement system needs to be developed for precise chemical structure research of melanin-like NPs. The complex kinetics in the polymerization process is expected to be solved by advanced calculation methods. For applications in vivo of melanin-like NPs, controllable distribution of organisms and on-off therapy are critical for biological safety and curative effect. Accordingly, more efforts based on these two parts should be carried out toward clinical transformation. NIR irradiation is an important factor for PTT based on melanin-like NPs. Present technology can control time and intensity of irradiation precisely but there is still much room for improvement in spatial accuracy. With the rapid development of optical fibers in the field of biomedicine, this updated technology is expected to contribute to the clinical transformation of PTT in the future. Another problem is that although the current targeted modification has improved the therapeutic effect of melanin-like NPs to a certain extent, uneven tumor tissue distribution and uncontrollable degradation in vivo limit their clinical application. To overcome these shortcomings, screening more specific targeting molecules is of great significance, which is conductive to achieving controllable biodistribution and enhanced local therapeutic effect. In addition, establishing a complete evaluation system including morphological characterization, chemical structure, polymerization mechanism, physicochemical properties, loading capacity, therapeutic effect, etc. will greatly promote the clinical applications of melanin-like NPs. Therefore, the future direction of melanin-like NPs in the field of cancer photothermal therapy is summarized as follows: (1) open up new ways to obtain nanoparticles (2) analysis of polymerization mechanism and chemical structure (3) enhanced curative effect in vivo (4) development of safe and efficient combination drug model.

In summary, the research of melanin-like NPs for photothermal therapy has achieved unprecedented progress in the past decade. It can be easily envisioned that with booming research of melanin-like NP, it will break through the current dilemma in the near future and accelerate the clinical transformation of photothermal anti-tumor therapy.
